# Area Deprivation and Postpartum Readmission Facility Location and Timing

**DOI:** 10.1001/jamanetworkopen.2024.4699

**Published:** 2024-04-03

**Authors:** Nicole A. Beckley, Sean G. Young, Jessica S. Hook, Donald D. McIntire, Elaine L. Duryea, Catherine Y. Spong, David B. Nelson

**Affiliations:** 1 Department of Obstetrics and Gynecology, University of Texas Southwestern Medical Center, Dallas; 2 Peter O’Donnell Jr. School of Public Health, University of Texas Southwestern Medical Center, Dallas; 3 Parkland Health, Dallas, Texas

## Abstract

This cohort study evaluates the role that community-level socioeconomic status plays in hypertension-related hospital readmission within 12 weeks after delivery.

## Introduction

Hypertension-related complications account for most postpartum readmissions.^1^ Although patient-level factors have been associated with postpartum readmission, the role of community-level social factors is less understood, and data ascertainment between hospitals is limited.^2^ We postulated that neighborhood-level factors may affect postpartum readmission facility location and timing. We aimed to ascertain whether neighborhood socioeconomic factors were associated with location and timing of readmission for hypertension-related readmission within 12 weeks after delivery.

## Methods

The Dallas Fort-Worth Hospital Council (DFWHC) database, a regional hospital dataset of individual patient data from more than 80 facilities in North Texas, was queried for inpatient readmission after delivery.^3^ The University of Texas Southwestern Medical Center’s Institutional Review Board and DFWHC approved this cohort study and waived informed consent because deidentified data were used. We followed the STROBE reporting guideline.

The DFWHC dataset includes deidentified data for all hospital encounters. Each patient is assigned a unique identifier that allows analysis of readmissions at any hospital system in the DFWHC region, and each encounter includes a patient’s GEO-ID, a numeric code that uniquely identifies a geographic area, facilitating linkage with geospatial datasets. Patients who had a live birth between January 1, 2014, and December 31, 2018, and had a readmission for a pregnancy-related hypertension disorder, as classified by *International Classification of Diseases, Ninth Revision* and *International Statistical Classification of Diseases and Related Health Problems, Tenth Revision* codes, within 12 weeks of delivery were included (eTable in Supplement 1).^4,5^ Only first hypertension readmissions were evaluated. Neighborhood socioeconomic disadvantage was assessed using the Texas-specific Area Deprivation Index (ADI), a validated measure of social and economic status reported at the Census block group level (ADI range: 1-10, with higher scores indicating disadvantages).^6^ The ADI is a multidimensional evaluation that uses factors, such as housing quality and income, to quantify the socioeconomic status of neighborhoods. Data from the DFWHC dataset and ADI were linked via the patient GEO-ID, with patients readmitted to a different hospital compared with those readmitted to the same hospital where they gave birth.

Statistical analysis was performed from September to October 2023 using GraphPad Prism 10.1.2 (GraphPad Software). χ^2^ was used to assess for association between hospital readmission location and ADI. Mann-Kendall test was used for trend analysis. Armitage trend test was used for cross-tabulation of same-vs-different readmission hospital for differing ADI. Two-sided *P* < .05 indicated statistical significance. Data are presented as medians with 95% CIs.

## Results

**Figure 1.**
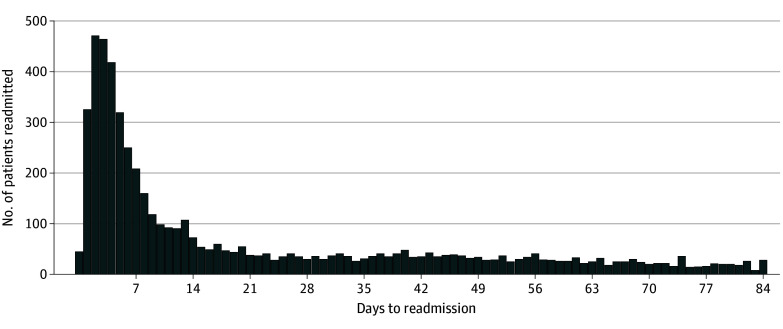
Number of Patients Readmitted for a Hypertension Disorder by Day, Up to 12 Weeks Post Partum

**Figure 2.**
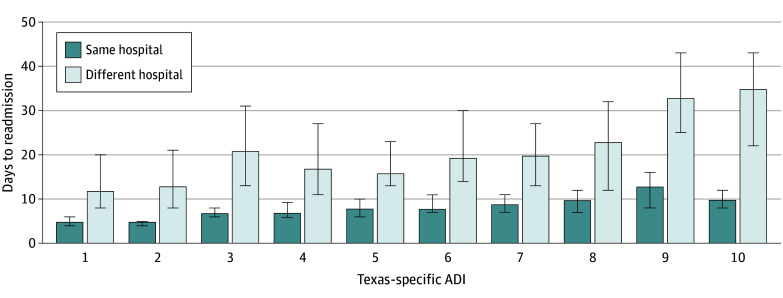
Days to Readmission Based on Area Deprivation Index (ADI) and Hospital Readmission Location Error bars represent 95% CIs.

Between 2014 and 2018, there were 475 865 deliveries, with 5471 (1.1%) patients (all females; mean [SD] age, 30.4 [6.3] years) having at least 1 readmission for a hypertension-related disorder within 12 weeks of delivery. Of these patients, 4037 (74%) were readmitted to the same hospital where they gave birth, and 1434 (26%) were readmitted to a different hospital. Most readmissions occurred within the first week post partum (Figure 1). Regardless of ADI, patients receiving care at a different hospital were readmitted later than those readmitted to the same hospital where they gave birth (20 [95% CI, 18-23] days vs 7 [95% CI, 7-8] days; *P* < .001). There was a trend of increasing rate of admission to a different hospital for increasing ADI. Percentage of admission to a different hospital increased from 21% (95% CI, 17%-26%) at an ADI of 1 to 28% (95% CI, 24%-32%) at an ADI of 10. Patients linked to an ADI of 10 presented to a different hospital 2.9 times later than patients linked to an ADI of 1 (35 [95% CI, 22-43] days vs 12 [95% CI, 8-20] days; *P* < .001) (Figure 2).

## Discussion

Approximately 1 in 4 postpartum patients requiring readmission for hypertension was admitted to a hospital other than their delivery hospital. Although limited by use of individual-level socioeconomic data, this study found that neighborhood-level socioeconomic disadvantage was associated with an increased likelihood of readmission to a different hospital and readmission at a later timing.

## Notes

Supplement 1. **eTable.** ICD-9 and ICD-10 Codes Used to Filter DFWHC Data

Supplement 2.
Data Sharing Statement
