# Supplementary material for: Area Deprivation and Postpartum Readmission Facility Location and Timing
Source: JAMA Netw Open. 2024 Apr 3;7(4):e244699. doi: 10.1001/jamanetworkopen.2024.4699 (PMC10993070; doi:10.1001/jamanetworkopen.2024.4699)
Supplement: Supplement 1. — eTable. ICD-9 and ICD-10 Codes Used to Filter DFWHC Data [file jamanetwopen-e244699-s001.pdf]

Supplemental Online Content

Beckley NA, Young SG, Hook JS, et al. Area deprivation and postpartum readmission facility location and timing. *JAMA Netw Open*. 2024;7(4):e244699. doi:10.1001/jamanetworkopen.2024.4699

eTable. ICD-9 and ICD-10 Codes Used to Filter DFWHC Data

This supplemental material has been provided by the authors to give readers additional information about their work.

© 2024 Beckley NA et al. *JAMA Network Open*

eTable. ICD-9 and ICD-10 Codes Used to Filter DFWHC Data

**Hypertension**	401.0, 401.1, 401.9, 642.00, 642.01, 642.03, 642.04, 642.10, 642.11, 642.13, 642.14, 642.20, 642.21, 642.23, 642.24, 642.30, 642.31, 642.32, 642.33, 642.34, 642.40, 642.41, 642.42, 642.43, 642.44, 642.50, 642.51, 642.52, 642.53, 642.54, 642.60, 642.61, 642.63, 642.64, 642.70, 642.71, 642.72, 642.73, 642.74, 642.90, 642.92, 642.94, 646.10, 646.11, 646.12, 646.13, 646.14, 646.20, 646.21, 646.22, 646.23, 646.24, O10.011, O10.012, O10.013, O10.019, O10.02, O10.03, O10.111, O10.112, O10.113, O10.119, O10.12, O10.13, O10.211, O10.212, O10.213, O10.219, O10.22, O10.23, O10.311, O10.312, O10.313, O10.319, O10.32, O10.33, O10.411, O10.412, O10.413, O10.419, O10.42, O10.43, O10.911, O10.912, O10.913, O10.919, O10.92, O10.93, O11.1, O11.2, O11.3, O11.4, O11.5, O11.9, O12.00, O12.01, O12.02, O12.03, O12.04, O12.05, O12.10, O12.11, O12.12, O12.13, O12.14, O12.15, O12.20, O12.21, O12.22, O12.23, O12.24, O12.25, O13.1, O13.2, O13.3, O13.4, O13.5, O13.9, O14.00, O14.02, O14.03, O14.04, O14.05, O14.10, O14.12, O14.13, O14.14, O14.15, O14.20, O14.22, O14.23, O14.24, O14.25, O14.90, O14.92, O14.93, O14.94, O14.95, O15.00, O15.02, O15.03, O15.1, O15.2, O15.9, O16.5, O16.9, I10

© 2024 Beckley NA et al. *JAMA Network Open*
