# Supplementary material for: Area Deprivation and Postpartum Readmission Facility Location and Timing
Source: JAMA Netw Open. 2024 Apr 3;7(4):e244699. doi: 10.1001/jamanetworkopen.2024.4699 (PMC10993070; doi:10.1001/jamanetworkopen.2024.4699)
Supplement: Supplement 2. — Data Sharing Statement [file jamanetwopen-e244699-s002.pdf]

Data Sharing Statement

Beckley. Area Deprivation and Postpartum Readmission Facility Location and Timing. *JAMA Netw Open*. Published April 03, 2024. doi:10.1001/jamanetworkopen.2024.4699

Data
Data available: No
